# Toll-like receptors and their role in the pathogenesis of myasthenia gravis: a comprehensive review

**DOI:** 10.3389/fimmu.2025.1624957

**Published:** 2025-12-19

**Authors:** Xiaoxiao Zheng, Ling Li, Hongyue Ma, Mingxia Zhu, Xiuli Li, Xinhong Feng

**Affiliations:** Department of Neurology, Beijing Tsinghua Changgung Hospital, School of Clinical Medicine, Tsinghua Medicine, Tsinghua University, Beijing, China

**Keywords:** myasthenia gravis, toll-like receptors, cytokine release, therapeutic targets, autoimmune neuromuscular disorders

## Abstract

Myasthenia gravis (MG) is a chronic autoimmune neuromuscular disorder marked by autoantibody-mediated dysfunction at the neuromuscular junction, resulting in fluctuating muscle weakness. The pathogenesis of MG involves a complex interplay between genetic predisposition, environmental factors, and immune system dysregulation. Among these, the innate immune system, particularly Toll-like receptors (TLRs), has emerged as a critical player in disease progression by influencing both innate and adaptive immunity. TLRs are a family of pattern recognition receptors (PRRs) that detect pathogen-associated molecular patterns (PAMPs) and damage-associated molecular patterns (DAMPs), triggering immune responses. Dysregulation of TLRs expression and signaling in MG has been implicated in chronic inflammation, breakdown of immune tolerance, and activation of autoreactive T and B cells. Overexpression of specific TLRs, such as TLR4 and TLR9, has been reported in MG patients, particularly in thymic tissues and peripheral immune cells, correlating with increased pro-inflammatory cytokine production and autoantibody generation. These aberrant responses contribute to the autoimmune cascade that underlies MG. Emerging evidence highlights the therapeutic potential of targeting TLRs pathways in MG. Strategies include using TLRs antagonists, modulating downstream signaling pathways, and leveraging epigenetic regulators to normalize TLRs activity. This review examines the role of TLRs in MG by exploring their expression profiles, their involvement in inflammatory signaling pathways, their impact on the adaptive immune system, and their potential as therapeutic targets. A better understanding of the role of TLRs in MG pathogenesis could open new avenues for modulating immune responses and precision therapies targeting the innate immune system.

## 1 Introduction

Myasthenia Gravis (MG) is an autoimmune neuromuscular disorder characterized by skeletal muscle weakness and fatigue, resulting from impaired transmission at the neuromuscular junction (NMJ) (1). The disease is mediated by autoantibodies targeting components of the NMJ, particularly the acetylcholine receptor (AChR), muscle-specific kinase (MuSK), and low-density lipoprotein receptor-related protein 4 (LRP4) (2). These autoantibodies cause both functional impairment and structural damage to the NMJ. MG presents heterogeneously, ranging from ocular symptoms to life-threatening respiratory failure (myasthenic crisis) (3). Thymic involvement is common, especially in cases with thymic hyperplasia or thymomas, indicating its role in disease pathogenesis (4). The development of MG involves a complex interaction between genetic predisposition (5), environmental factors (6), and immune system dysregulation (7). Both innate and adaptive immune systems contribute to the breakdown of self-tolerance (8). While the adaptive immune system has been extensively studied in MG, recent research highlights the role of innate immune components, particularly Toll-like receptors (TLRs), in the disease process.

TLRs are critical pattern recognition receptors (PRRs) in the innate immune system, responsible for detecting pathogen-associated molecular patterns (PAMPs) and damage-associated molecular patterns (DAMPs) (9, 10). They are expressed on immune and non-immune cells, such as dendritic cells (DCs), macrophages, B cells, T cells, and epithelial cells (11). Upon activation, TLRs trigger signaling cascades that produce pro-inflammatory cytokines, chemokines, and type I interferon (IFN), initiating immune responses to infections. Dysregulated TLRs activation has been implicated in several autoimmune diseases, including systemic lupus erythematosus (SLE) (12), rheumatoid arthritis (RA) (13), and type 1 diabetes (T1D) (14). In MG, altered TLRs expression and function may contribute to autoimmunity by enhancing the activation of autoreactive T and B cells, promoting autoantibody production, and sustaining chronic inflammation (7). This review provides a comprehensive analysis of the involvement of TLRs in MG pathogenesis, including their expression patterns, signaling pathways, and interactions with other immune components. A deeper understanding of TLRs involvement in MG pathogenesis could pave the way for novel precision therapies aimed at modulating innate immunity.

## 2 Structure, function, and signaling of TLRs

### 2.1 Overview of TLRs family

TLRs are a conserved family of receptors that play a crucial role in the innate immune system by recognizing PAMPs and DAMPs (9). TLRs were first identified in Drosophila and are now recognized as key components of vertebrate innate immunity (15). Ten TLRs (TLR1–TLR10) have been identified in humans (16), and they are expressed on a variety of immune and non-immune cells, including DCs, macrophages, neutrophils, epithelial cells, and B and T lymphocytes (17, 18). Based on their subcellular localization, TLRs are classified as: Surface-Expressed TLRs (e.g., TLR1, TLR2, TLR4, TLR5, TLR6, TLR10) detect microbial components like lipopolysaccharides (LPS) (19) and flagellin (20). Intracellular TLRs (e.g., TLR3, TLR7, TLR8, TLR9) recognize nucleic acids from viruses and damaged cells (21). For instance, TLR4 recognizes LPS from Gram-negative bacteria, TLR1/6 detects bacterial tri-acylated proteins, and TLR9 senses unmethylated CpG DNA (19, 22, 23). Upon ligand binding, TLRs undergo conformational changes that lead to the recruitment of adaptor proteins, such as myeloid differentiation primary response 88 (MyD88) and toll/interleukin-1 receptor (TIR)-domain-containing adapter-inducing interferon-β (TRIF). These adaptor proteins initiate downstream signaling cascades involving kinases like interleukin-1 receptor-associated kinases (IRAKs) and TNF receptor-associated factor 6 (TRAF6), ultimately activating transcription factors such as nuclear factor kappa-light-chain-enhancer of activated B cells (NF-κB), activator protein 1 (AP-1), and interferon regulatory factors (IRFs) (24). The activation of these transcription factors results in the production of inflammatory cytokines, chemokines, and type I IFN, which are essential for mounting effective immune responses (9).

### 2.2 Structural characteristics of TLRs

**Figure 1:**
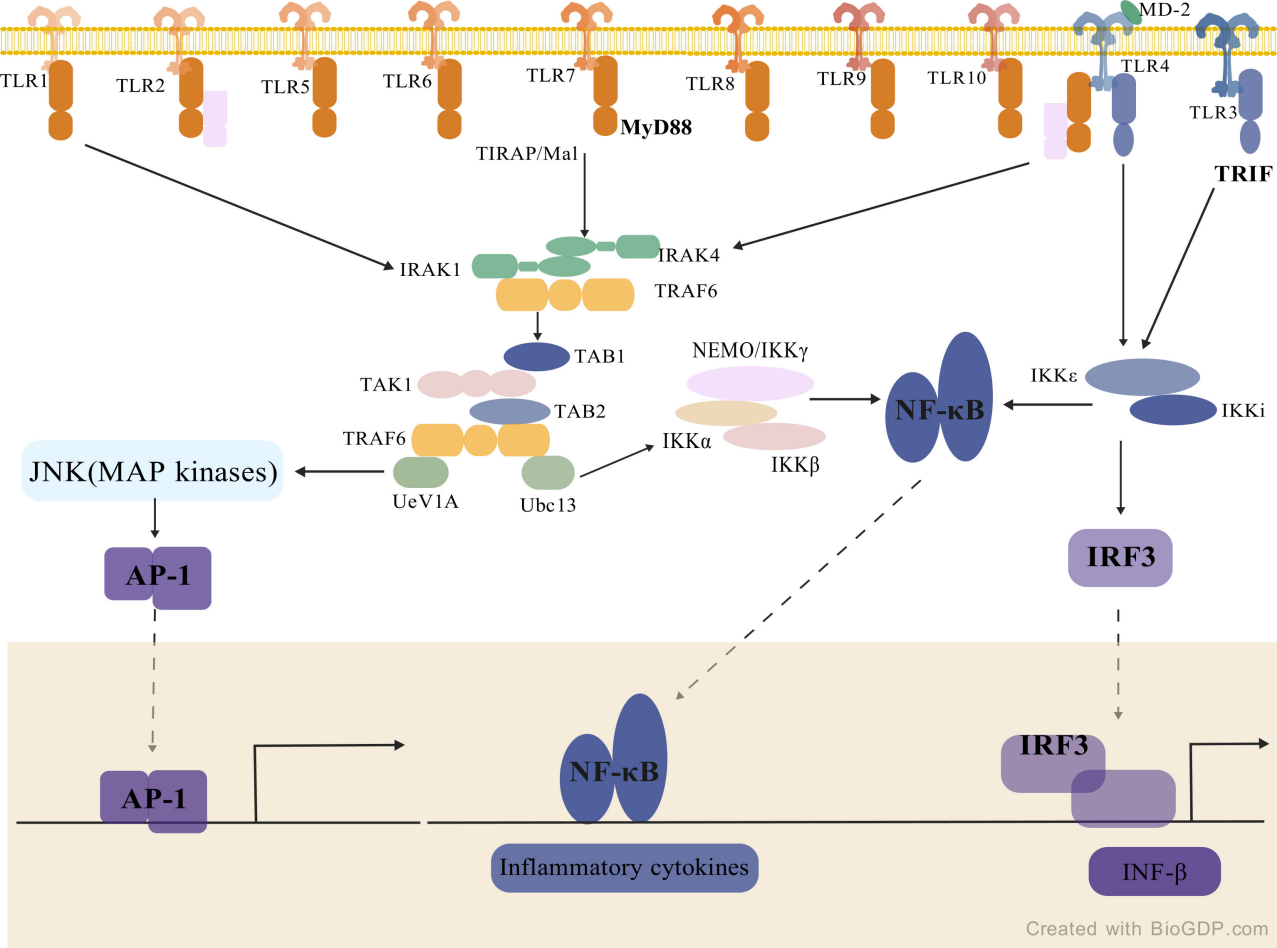
TIRs domain-containing adaptors and TLRs signaling (created with BioGDP.com) (26). MyD88 is an essential TIRs domain-containing adaptor for the induction of inflammatory cytokines via all the TLRs. TIRAP/Mal is a second TIR domain-containing adaptor that specifically mediates the MyD88-dependent pathway via TLR2 and TLR4. MyD88 binds to the cytoplasmic portion of TLRs through interaction between individual TIR domains. Upon stimulation, IRAK-4, IRAK-1, and TRAF6 are recruited to the receptor, which induces association of IRAK-1 and MyD88 via the death domains. IRAK-4 then phosphorylates IRAK-1. Phosphorylated IRAK-1, together with TRAF6, dissociates from the receptor and then TRAF6 interacts with TAK1, TAB1, and TAB2. The complex of TRAF6, TAK1, TAB1, and TAB2 further forms a larger complex with Ubc13 and Uev1A, which induces the activation of TAK1. Activated TAK1 phosphorylates the IKK complex, consisting of IKKα, IKKβ, and NEMO/IKKγ, and MAP kinases, such as JNK, and thereby induces the activation of the transcription factors NF-κB and AP-1, respectively. In the TLR4- and TLR3-mediated signaling pathways, a MyD88-independent pathway exists that leads to activation of IRF-3 via TBK1 and IKKϵ/IKKi. The TIR domain-containing adaptor TRIF mediates this MyD88-independent pathway. TLRs, toll-like receptors; MyD88, myeloid differentiation primary response 8; TIR, toll/interleukin-1 receptor; TRIF, TIR-domain-containing adapter-inducing interferon-β; TIRAP/Mal, TIR domain-containing adaptor protein; IRAKs, interleukin-1 receptor-associated kinases; TRAF6, TNF receptor-associated factor 6; TAK1, TGF-β activated kinase 1; TAB1, TGF-β activated kinase 1 binding protein 1; TAB2, TGF-β activated kinase 1 binding protein 1; NF-κB, nuclear factor kappa-light-chain-enhancer of activated B cells; AP-1, activator protein 1; IRFs, interferon regulatory factors; IKK, inhibitor of nuclear factor kappa-B kinase; JNK, c-Jun N-terminal kinase; MAPK, mitogen-activated protein kinase; TBK1, TANK-binding kinase 1; IFN-β, interferon beta.

TLRs, as type I transmembrane proteins, exhibit a structure-function relationship critical for their immunological roles. These receptors comprise three characteristic domains: 1) Leucine-rich repeat (LRR) extracellular domain: This horseshoe-shaped module mediates ligand specificity, recognizing both microbial components (e.g., LPS, nucleic acids) and host-derived molecules (e.g., heat shock proteins). Structural plasticity of LRR domains underlies the ligand selectivity among TLRs family members; 2) Transmembrane domain: Beyond membrane anchoring, its hydrophobic α-helices participate in subcellular localization regulation; 3) Cytoplasmic TIR domain: This effector module initiates signal transduction by recruiting adaptor proteins including MyD88 and TRIF (25), as illustrated in **Figure 1**. Notably, conformational changes in TIR domains determine the selective recruitment of downstream adaptors. This modular architecture enables TLRs to maintain ligand discrimination while achieving signaling diversification (25). Ligand-induced conformational changes in LRR domains are transmitted through transmembrane segments, ultimately triggering immune responses via TIR domain activation (27).

### 2.3 TLRs family members and their ligands

1) Surface-Expressed TLRs are expressed on the plasma membrane and primarily recognize microbial components from bacteria, fungi, and parasites; 2) Intracellular TLRs are localized in endosomal compartments and primarily recognize nucleic acids from viruses, bacteria, and damaged host cells (9). They are all summarized in **Table 1**.

**Table 1:** Classification, ligands, and functions of TLRs based on subcellular localization.

TLR class	TLR member	Ligands recognized	Key features	
Surface-Expressed TLRs	TLR1	Tri-acylated lipoproteins (with TLR2)	Forms heterodimers with TLR2 to recognize bacterial lipoproteins	(22)
	TLR2	Lipoteichoic acid, peptidoglycan, zymosan (fungal components)	Partners with TLR1 or TLR6 to detect diverse bacterial/fungal components	(22)
	TLR4	LPS from Gram-negative bacteria	Requires co-receptors CD14 and MD-2 for LPS recognition	(19)
	TLR5	Bacterial flagellin	Detects flagella proteins from motile bacteria	(20)
	TLR6	Di-acylated lipoproteins (with TLR2)	Cooperates with TLR2 to sense bacterial lipoproteins	(22)
	TLR10	Unknown (possibly host-derived peptides)	Role in immune regulation; exact ligands unclear.	(28)
Intracellular TLRs	TLR3	dsRNA	Detects viral dsRNA, triggering antiviral responses	(21)
	TLR7	ssRNA from viruses	Highly expressed in pDCs	(29)
	TLR8	ssRNA from viruses	More active in monocytes and macrophages	(29)
	TLR9	Unmethylated CpG DNA (bacterial/viral genomes)	Recognizes microbial DNA in endosomal compartments	(23)

TLRs, toll-like receptors; LPS, lipopolysaccharides; dsRNA, double-stranded RNA; ssRNA, single-stranded RNA; pDCs, plasmacytoid dendritic cells.

### 2.4 TLRs signaling pathways

TLRs mediate immune responses through two principal signaling cascades, with differential activation mechanisms dictated by ligand specificity and receptor subcellular localization. In the MyD88-dependent pathway, utilized by all TLRs except TLR3, the adaptor protein MyD88 recruits the inhibitor of nuclear factor kappa-B kinase (IKK) complex and mitogen-activated protein kinase (MAPK) cascades, leading to nuclear translocation of transcription factors NF-κB and AP-1 (30). This pathway predominantly induces proinflammatory cytokines (e.g., tumor necrosis factor alpha [TNF-α], IL-6, and IL-1β), playing a pivotal role in antibacterial responses. In contrast, the TRIF-dependent pathway is selectively activated by TLR3 and partially by TLR4 through the adaptor TRIF, which initiates the TANK-binding kinase 1 (TBK1)-IRF3/7 axis to drive type I IFN (IFN-α/β) production (31). Notably, TLR4’s capacity to engage both pathways enables dual functionality in combating bacterial and viral infections. This signaling dichotomy carries profound biological implications: while the MyD88 pathway mediates rapid inflammatory responses, the TRIF pathway establishes antiviral states, together constituting a multilayered defense system against diverse pathogens (10).

### 2.5 Expression patterns of TLRs

TLRs exhibit a cell-type-specific expression pattern that aligns with their immunoregulatory functions, being ubiquitously distributed across both immune and non-immune cell populations. Within the immune system, constitutive TLRs expression is observed in antigen-presenting cells (APCs) (e.g., DCs, macrophages, and B cells, which specialize in processing and presenting antigens to T cells) and myeloid lineages (monocytes, neutrophils), enabling rapid initiation of innate immune responses upon pathogen detection (17, 18). Notably, the subset-specific TLRs expression in natural killer (NK) cells and lymphocytes (B and T cells) correlates with their functional specialization in adaptive immunity (32–34). Among non-immune cells, TLRs expression in fibroblasts, epithelial cells, endothelial cells, and thymic epithelial cells (TECs) underscores their roles in barrier defense and tolerance maintenance (17, 18). Particularly, aberrant TLRs expression in TECs has been implicated in the breakdown of central tolerance during autoimmunity (35). This pan-lineage expression profile suggests TLRs serve pleiotropic functions beyond pathogen recognition: while primarily mediating inflammatory responses in immune cells, they predominantly contribute to tissue-specific immunomodulation in non-immune compartments (36).

### 2.6 TLRs regulation

Precise regulation of TLRs activity is essential for maintaining immune homeostasis, with dysregulation implicated in chronic inflammation and autoimmunity. Multiple negative regulatory mechanisms operate at molecular levels: (i) A20 dampens NF-κB signaling through deubiquitination of key intermediates like TRAF6 (37); (ii) suppressors of cytokine signaling (SOCS) proteins terminate TLRs responses by blocking janus kinase-signal transducer and activator of transcription (JAK-STAT) pathways and promoting signalosome degradation (38); (iii) aberrant recognition of endogenous ligands (e.g., heat shock proteins, high mobility group box 1 [HMGB1], and self-DNA) may break immune tolerance, particularly evidenced in autoimmune diseases like SLE (39). TLRs dysregulation drives autoimmunity through three interconnected tiers: enhanced sensitivity to self-antigens via TLRs overexpression on immune cells, erroneous classification of DAMPs as danger signals, and sustained activation of downstream effectors (NF-κB/IRF) that collectively establish a proinflammatory milieu favoring autoreactive lymphocyte activation (36, 40). This multilevel dysregulation has been experimentally validated in thymic tissues and peripheral immune cells from MG patients.

### 2.7 TLRs in autoimmune diseases

TLRs are pivotal in recognizing microbial and endogenous ligands and bridging innate and adaptive immunity. While they are crucial for immune defense, dysregulated TLRs activity plays a significant role in the pathogenesis of autoimmune diseases, including MG (9). Emerging evidence indicates that aberrant TLRs expression and signaling are involved in multiple autoimmune diseases. For instance, in SLE (12), TLR7 and TLR9 recognize self-nucleic acids, triggering type I IFN production and autoantibody generation. In RA (13), upregulation of TLR2 and TLR4 in synovial tissues perpetuates chronic inflammation, whereas in T1D (14), dysregulated TLRs signaling in pancreatic β-cells and immune cells exacerbates autoimmune destruction. These findings highlight the dual role of TLRs in autoimmunity—serving as both guardians of immune surveillance and drivers of pathological responses. Thus, targeted modulation of TLRs pathways may represent a promising therapeutic strategy for these diseases.

## 3 Expression and regulation of TLRs in MG

TLRs play a dual role in MG: TLRs are implicated in dysregulation of thymic and peripheral immune responses, contributing to the development of autoimmunity. Aberrant TLRs activation in DCs and macrophages promotes inflammatory cytokine production and antigen presentation, facilitating autoantibody production (7). Overexpression of TLRs in the thymus may contribute to the breakdown of central tolerance and the survival of autoreactive T cells.

### 3.1 Altered TLRs expression in MG

**Figure 2:**
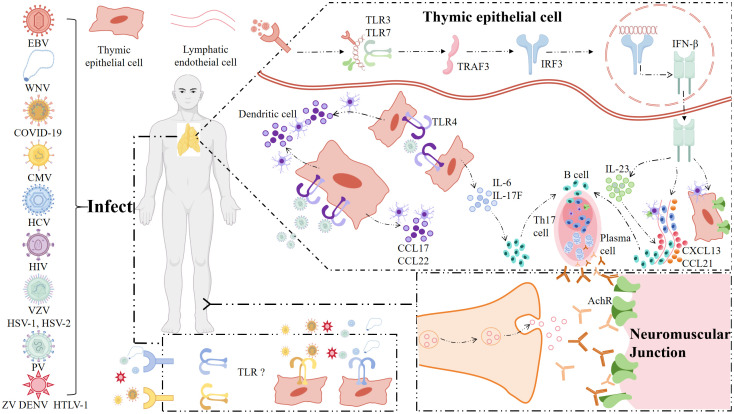
The invading pathogens participates in the pathogenesis in MG. The invading pathogens trigger the aberrant activation of TLRs pathways in the thymus. **(a)** EBV binds to TLR3 or TLR7, resulting in the overproduction of pro-inflammation cytokines such as IFN-β and chemokines to recruit peripheral B cells and Th17 cells to generate GCs in thymus. IFN-β also promotes TECs to express AChR and uptaken by APCs, leading to the autosensitization against AChR and the production of autoantibodies; **(b)** TLR4 pathway is activated by poliovirus, resulting in the expression of chemokines to attract DCs and the production of Th17-related cytokines to alter the effector T cell/regulatory T cell balance; **(c)**. The AChR antibodies play their role at both thymus and peripheral NMJ via activating the classical complement pathway causing damage to the TECs and postsynaptic membrane, respectively. Type I IFN, which is produced by pDCs in response to autoimmune complexes containing RNA and DNA recognized by TLR7 and 9 acts as a potent amplifier of the autoimmune response. TLRs-independent recognition of self nucleic acids by cytosolic RNA and DNA sensors may also play a role in the generation of autoimmune responses. TLRs, toll-like receptors; EBV, Epstein-Barr virus; IFN-β, interferon beta; Th17, T helper 17; GCs, germinal centers; TECs, thymic epithelial cells; AChR, acetylcholine receptor; APCs, antigen-presenting cells; DCs, dendritic cells. (created with BioGDP.com) (26).

Evidence suggests that TLRs expression is dysregulated in MG, contributing to both systemic and localized inflammation: 1) TLRs expression in immune cells: Studies have demonstrated altered expression of TLRs in various immune cells of MG patients. For example, defective microRNA-146a (miR-146a) expression contributed to persistent TLRs activation in peripheral blood mononuclear cells (PBMCs) from MG patients (41), suggesting a heightened state of immune activation. Additionally, DCs and macrophages from MG patients exhibit upregulated TLRs expression (42), which may enhance their ability to present autoantigens and activate autoreactive T cells; 2) TLRs expression in thymus: The thymus plays a central role in MG pathogenesis, particularly in patients with thymic hyperplasia or thymomas. Altered TLRs expression in the thymus may contribute to defective negative selection of autoreactive T cells and the development of autoimmunity. Increased expression of TLR4 (36), TLR7, and TLR9 (42) has been reported in TECs from MG patients, which may promote local inflammation and the survival of autoreactive lymphocytes. The thymus plays a central role in the pathogenesis of MG, and thymic epithelial cells (TECs) are the key architects of this process. TECs are functionally and anatomically divided into two main subsets: cortical TECs (cTECs) and medullary TECs (mTECs), which work in concert to establish central immune tolerance (43). cTECs are responsible for the “positive selection” of thymocytes by presenting self-antigenic peptides bound to MHC molecules, ensuring the survival of T cells capable of recognizing self-MHC. Subsequently, mTECs perform the critical task of “negative selection” by expressing a vast repertoire of tissue-restricted self-antigens (including the key MG autoantigen, the AChR) under the control of the Autoimmune Regulator (AIRE), thereby eliminating self-reactive T cell clones (44). In MG, the aberrant expression of TLRs in TECs directly disrupts this finely tuned tolerogenic machinery. Studies have demonstrated overexpression of TLR4, TLR7, and TLR9 in TECs from MG patients (36, 42). This dysregulation can contribute to autoimmunity through several interconnected mechanisms. Aberrant TLR activation in TECs (e.g., by viral infections or endogenous DAMPs) drives the production of copious pro-inflammatory cytokines (such as type I IFN, IL-6, and TNF-α) and chemokines (40). This local inflammatory milieu fundamentally alters thymic homeostasis, transforming a site of tolerance into one of immune activation. It not only directly supports the survival and proliferation of autoreactive lymphocytes but also recruits peripheral immune cells (like B cells and mature dendritic cells) into the thymus, facilitating the formation of ectopic germinal centers, a hallmark of MG thymic pathology (45). TLR signaling, particularly pathways involving type I IFN, has been shown to significantly upregulate the expression of self-antigens (such as AChR) in mTECs (46). This “over-exposure” during negative selection may paradoxically lead to the activation rather than the deletion of some autoreactive T cell clones that would otherwise have escaped due to insufficient antigen avidity. Concurrently, the inflammatory environment can impair the function of the AIRE, a transcription factor critical for the promiscuous expression of tissue-specific antigens in mTECs. Compromised AIRE function results in an incomplete presentation of the self-antigen repertoire, allowing certain autoreactive T cell clones to evade deletion (47). The combined effects of persistent inflammatory signaling and altered self-antigen presentation ultimately compromise the efficiency of negative selection. Autoreactive T cells, including those specific for AChR, fail to be effectively eliminated and thus escape to the periphery (48). Once in the periphery, these “rogue” cells can be activated, initiating the autoimmune attack on the neuromuscular junction. In conclusion, dysregulated TLR signaling in TECs serves as a critical bridge connecting innate immune activation to the breakdown of adaptive immune tolerance in MG. By transforming the thymus from a cradle of tolerance into a site of autoimmunity initiation, it plays a foundational role in the pathogenesis of the disease; 3) NMJ: although direct evidence is limited, TLRs may indirectly influence NMJ inflammation by amplifying systemic autoimmune responses, as illustrated in **Figure 2**.

The relationship between TLR7/9 overexpression and EBV-positive thymic inflammation involves a sophisticated immunological cascade. plasmacytoid dendritic cells (pDCs), which highly express TLR7 and TLR9, are recruited to the thymus and play a central role in this process. Upon recognition of EBV-derived nucleic acids, these pDCs become activated and produce substantial amounts of type I IFN, particularly IFN-α, establishing a characteristic interferon signature within the thymic microenvironment (40). This type I IFN response acts as a potent immunological adjuvant that promotes the maturation of conventional dendritic cells, enhances antigen presentation to autoreactive T cells, and supports B-cell activation and differentiation. Critically, this sustained inflammatory milieu facilitates the formation and maintenance of ectopic germinal centers within the thymus. These structural abnormalities provide a survival niche for autoreactive B cells, enabling their local proliferation and affinity maturation, ultimately leading to the production of pathogenic autoantibodies against the acetylcholine receptor (42, 45). This coordinated mechanism, driven by TLR7/9 signaling in pDCs, effectively transforms the thymus from a site of self-tolerance induction into a hub for autoimmune amplification.

In the study of Wang et al., significantly different expression of TLRs between MG patients and the healthy controls has been observed, and expression of TLR1, 6, and 10 mRNA were significantly downregulated while mRNA of TLR2, 3, 4, 5, 8, and 9 were significantly upregulated (49). It is hypothesized that downregulated TLRs may play protective roles in MG while the upregulated TLRs promote the onset of the disease.

They hereby observed universal abnormalities of the TLRs (except TLR7) while only TLR9 was positively related to the Quantitative MG (QMG) scores of MG patients (49). EN101, a TLR9-specific ligand, was administered to patients with MG. After 2 and 4 weeks, patients with all doses show a decrease in QMG scores, with a greater response to higher doses (50). It is consistent with the phenomenon that the induction of experimental autoimmune myasthenia gravis (EAMG) models requires the combination of antigen or AChR peptide with complete Freund’s adjuvant, which respectively activates different TLRs including TLR4, TLR7, TLR8 and TLR9 (58). TLR7 serves as the sensor of infection with single-stranded RNA (ssRNA) viruses and is able to assist the activation of TLR9 (29). Both of them were over-expressed in TECs and APCs of nonthymomatous MG thymuses with Epstein-Barr virus (EBV) (42). The proinflammatory effects of TLR7/TLR9 were mediated by the overexpression of type I IFN especially IFN-β and the abnormal proliferation of B cells (40). Moreover, the gene analysis showed significant overexpression of TLR5 in MG thymoma and TLR10 in hyperplasia thymus, providing the genetic evidence for the correlation between these TLRs and MG (51). However, more studies need to be done to identify their exactly molecular mechanism in the pathogenesis of MG (7).

Beyond the association between TLR9 expression and QMG scores, a broader body of evidence from both experimental models and clinical studies underscores a significant correlation between hyperactive TLR signaling and MG severity. Experimentally, the direct activation of specific TLRs potently exacerbates disease. TLR3 signaling, triggered by viral dsRNA mimics, aggravates EAMG by promoting a pro-inflammatory Th1 and Th17 response, leading to increased anti-AChR antibody titers and more severe muscle weakness (52). Similarly, TLR4 activation enhances the activation of AChR-specific T and B cells, and TLR4 signaling through the MyD88 pathway is essential for the development of EAMG, driving the differentiation of T follicular helper cells and germinal center B cells critical for high-affinity autoantibody production (53, 54). Clinically, this hyper-responsiveness is mirrored in MG patients. Blood mononuclear cells from patients exhibit exaggerated production of inflammatory cytokines like IFN-γ, IL-4 and transforming growth factor beta (TGF-β) upon stimulation, correlating with a systemic pro-inflammatory state (57). The thymus, a key site of pathogenesis, shows significant overexpression of TLRs, which is believed to drive the activation of autoreactive lymphocytes within ectopic germinal centers, a hallmark of active disease (7, 55). Furthermore, genetic predisposition plays a role (56). These key experimental and clinical pieces of evidence, highlighting the role of TLR signaling in MG pathogenesis and severity, are summarized in **Table 2**. Collectively, this evidence positions TLRs not merely as initiators but as amplifiers of the autoimmune response, whose activity is positively correlated with disease severity and ongoing inflammation, thereby solidifying their relevance as therapeutic targets for modulating clinical outcomes.

**Table 2:** Summary of key TLR alterations in MG and their clinical/experimental evidence.

TLR member	Alterations in expression/function in MG	Study type	Association with disease severity and mechanisms	Reference(s)
TLR2	Upregulated mRNA and protein expression; enhanced monocyte responsiveness in patients.	Clinical Study	Associated with a systemic pro-inflammatory state; expression levels are higher in patients with active disease.	(49, 57)
TLR3	Upregulated mRNA expression.	Experimental, Clinical	In EAMG, its activation exacerbates disease severity by promoting Th1/Th17 responses and increasing antibody titers. Expression on B cells correlates with disease activity in patients.	(49, 52)
TLR4	Upregulated mRNA and protein expression in thymic epithelial cells and peripheral immune cells.	Experimental, Clinical	In EAMG, its activation enhances AChR-specific lymphocyte responses, worsening disease. In patients, high TLR4 expression on monocytes correlates with enhanced inflammation and disease activity.	(36, 49, 53)
TLR5	Gene expression significantly upregulated in thymoma.	Genetic/Clinical	Genetic evidence links it to thymoma-associated MG; the exact molecular mechanism requires further elucidation.	(51)
TLR7	Overexpressed in the thymus (particularly in germinal center B cells and epithelial cells) and peripheral immune cells.	Experimental, Clinical	Essential for EAMG development, driving Tfh cell differentiation and autoantibody production. Thymic overexpression in patients links to viral sensing and local inflammation; its polymorphisms influence disease risk and severity.	(40, 42)
TLR8	Overexpressed in the thymus.	Clinical Study	Associated with thymic pathology, postulated to contribute to local immune activation.	(21, 58)
TLR9	Upregulated mRNA expression; altered in both thymus and peripheral blood.	Experimental, Clinical	Expression levels positively correlate with patient QMG scores; ligand-based therapy improves scores, providing a functional link to severity. Plays a complex, context-dependent role in EAMG.	(40, 42)
TLR10	Gene expression upregulated in hyperplastic thymus.	Genetic/Clinical	Genetic evidence suggests an association with hyperplastic thymus-related MG; specific function requires investigation.	(16)

### 3.2 TLRs-driven inflammatory cytokine responses in MG

TLRs activation leads to the production of pro-inflammatory cytokines such as IL-1β, IL-6, and TNF-α, which are elevated in MG patients. Below, we discuss how each of these cytokines is involved in the immune dysregulation seen in MG: 1) IL-1β is a potent pro-inflammatory cytokine that is involved in initiating and amplifying immune responses. In MG, IL-1β contributes to the induction of T helper 1 (Th1) and T helper 17 (Th17) responses, which are known to drive autoimmunity. IL-1β also enhances the production of other cytokines, including TNF-α and IL-6, perpetuating the inflammatory cascade (59). Additionally, IL-1β plays a role in activating APCs such as DCs, which can drive the differentiation of autoreactive T cells; 2) IL-6 is a pleiotropic cytokine that plays a critical role in the differentiation and survival of B cells, plasma cells, and Th17 cells, all of which are involved in the autoimmune processes in MG. IL-6 promotes the survival and expansion of autoreactive B cells that produce pathogenic autoantibodies against the AChR, thereby contributing to the neuromuscular dysfunction seen in MG patients (60). Elevated IL-6 levels are also associated with disease activity and may correlate with disease severity; 3) TNF-α is a key cytokine in inflammatory processes and is involved in the activation of both innate and adaptive immune responses. In MG, TNF-α contributes to the chronic inflammation and immune cell activation that sustain the autoimmune response. It can enhance the activation and proliferation of autoreactive T and B cells, contributing to the production of autoantibodies (45). Additionally, TNF-α plays a role in the formation of ectopic germinal centers in the thymus, which are involved in the maturation of self-reactive lymphocytes (45).

In the pathogenesis of MG, proinflammatory cytokines IL-1β, IL-6 and TNF-α collectively disrupt immune tolerance and sustain autoimmune responses through multiple mechanisms (61). These cytokines exert their effects via several key pathways: First, they compromise immune tolerance by promoting the activation and proliferation of autoreactive T and B cells. IL-6 drives Th17 cell differentiation while TNF-α stimulates T-cell activation and differentiation, collectively maintaining abnormal immune responses against self-antigens such as the AChR. Second, IL-6 plays a pivotal role in supporting plasma cell survival and differentiation. The pathogenic autoantibody-producing plasma cells in MG patients depend on IL-6-mediated survival signals, and these antibodies directly interfere with neuromuscular transmission, leading to characteristic muscle weakness. Notably, chronic thymic inflammation represents another hallmark of MG. TNF-α and IL-1β not only promote ectopic germinal center formation within the thymus but also create a microenvironment conducive to aberrant selection of autoreactive T and B cells. These lymphoid follicle-like ectopic structures further amplify autoimmune responses. Most importantly, this cytokine network, driven by TLRs activation, establishes a self-perpetuating chronic inflammatory state. Persistently elevated levels of IL-1β, IL-6 and TNF-α create a microenvironment that favors sustained immune activation, resulting in continuous autoantibody production, lymphocyte activation and tissue damage at the NMJ, which explains the chronic progressive nature of MG.

### 3.3 Promotion of autoantibody production

In autoimmune diseases like MG, TLRs activation has been shown to contribute to the activation of autoreactive B cells and the subsequent production of pathogenic autoantibodies (62). These autoantibodies target self-antigens, such as the AChR at the NMJ, leading to muscle weakness and dysfunction, the hallmark of MG.

#### 3.3.1 TLRs expression and autoreactive B cell activation

In MG, the activation of TLRs has been linked to the activation and differentiation of autoreactive B cells. TLRs, especially TLR-4, TLR-7, and TLR-9, are expressed on various immune cells, including B cells (7). Under normal conditions, B cells play a role in producing antibodies as part of the adaptive immune response. However, in MG and other autoimmune diseases, aberrant activation of B cells by TLRs results in the production of autoantibodies (62).

#### 3.3.2 Pathogenesis of MG associated with viral infection

Although most MG cases lack an identified infectious trigger, case reports and serological evidence suggest that viral infections may act as environmental triggers initiating disease in genetically susceptible individuals. Though there was no specific link between pathogen infection and MG, several viral infections such as EBV, West Nile virus (WNV), coronavirus disease 2019 (COVID-19) (63, 64) have been anecdotally associated with MG onset, potentially through TLR activation serving as a trigger. A proposed mechanism linking viral infection, TLR activation, and MG pathogenesis is summarized in **Figure 2**. There is no evidence that this neurotropic virus causes morphological damage to AChR subunits or the NMJ. Rather, the defect in neuromuscular transmission appears to be mediated indirectly by host factors induced by virus. These endogenous TLRs ligands act as auto-adjuvants providing a stimulatory signal together with the pathogen when the pathogen breaks into the thymic epithelial cell (42).

#### 3.3.3 TLRs-mediated B cell activation

The binding of specific ligands (such as DNA or RNA) to TLRs on B cells can promote their activation (62). Once activated, B cells undergo clonal expansion and differentiate into plasma cells, which are responsible for producing antibodies (65). In the context of MG, these antibodies may recognize self-antigens, particularly the AChR, leading to autoimmunity. TLRs signaling, through pathways involving NF-κB and other transcription factors, enhances the survival and differentiation of autoreactive B cells, making them more likely to produce pathogenic autoantibodies.

### 3.4 T cell activation and differentiation

TLRs are crucial components of the innate immune system, playing a central role in the detection of pathogens and initiating immune responses. TLRs not only activate innate immune cells like DCs and macrophages (66) but also influence the adaptive immune response by modulating T cell differentiation and function (67). One of the most significant impacts of TLRs signaling on T cells is its potential to skew the balance between different T helper (Th) cell subsets, particularly towards pro-inflammatory Th17 cells (68), while potentially reducing the population or function of regulatory T cells (Tregs). This shift can disrupt immune tolerance and contribute to autoimmune diseases. In MG, TLR-driven Th17 responses are implicated in thymic inflammation and autoantibody production, highlighting their relevance to disease pathogenesis (40).

#### 3.4.1 TLRs signaling and Th17 cell differentiation

Th17 cells are a subset of CD4+ T cells characterized by the production of the pro-inflammatory cytokine IL-17 (69), which plays a key role in host defense against pathogens, especially at mucosal surfaces. However, when Th17 cells are inappropriately activated or expanded, they can contribute to chronic inflammation and autoimmunity. TLRs signaling is known to influence the differentiation and function of Th17 cells through several mechanisms: 1) TLRs Ligands and Th17 Polarization. TLRs activation can drive the differentiation of naïve T cells into Th17 cells in the presence of specific cytokines like IL-6 and TGF-β (68). TLRs, particularly TLR-2, TLR-4, TLR-7, and TLR-9, play a key role in promoting the Th17 differentiation process. For instance, in the presence of TLR ligands, such as LPS, peptidoglycan, or viral RNA, DCs and macrophages are activated to secrete IL-6, which is crucial for Th17 differentiation. IL-6 synergizes with TGF-β to enhance the expression of the transcription factor retinoic acid receptor-related orphan receptor gamma t (RORγt), which is essential for Th17 development; 2) Pro-inflammatory Cytokine Production. TLRs signaling activates NF-κB (70) and MAPK pathways (71), which in turn promote the production of pro-inflammatory cytokines such as IL-6, TNF-α, and IL-1β. These cytokines not only recruit and activate Th17 cells but also enhance their stability and function. Elevated IL-6 levels, in particular, have been associated with an increased Th17 response and a subsequent reduction in Treg function. Thus, TLRs activation promotes a pro-inflammatory environment conducive to Th17-driven inflammation; 3) TLRs and Th17 in Autoimmunity. In autoimmune diseases, the dysregulation of Th17 cell responses, often driven by TLRs signaling, contributes to tissue damage and chronic inflammation. Diseases such as RA, multiple sclerosis (MS), and inflammatory bowel disease are associated with the expansion of Th17 cells (72), which produce IL-17 and other inflammatory mediators that cause tissue destruction. TLRs-induced Th17 differentiation is thought to play a central role in the pathogenesis of these diseases.

#### 3.4.2 TLRs signaling and Tregs

Tregs, particularly the CD4+ CD25+ forkhead box P3 (Foxp3)+ subset, are essential for maintaining immune tolerance and preventing autoimmune responses (73). Tregs suppress the activity of autoreactive T cells, thereby preventing excessive immune responses and tissue damage. However, TLRs signaling can also affect Treg differentiation and function.

TLRs signaling, especially through the activation of pro-inflammatory cytokines like IL-6 and TNF-α, can impair Treg function. In particular, IL-6 has been shown to inhibit Treg differentiation and function by promoting the differentiation of naïve T cells into Th17 cells (74). This imbalance between Th17 and Tregs contributes to a loss of immune tolerance and the development of autoimmune conditions. Additionally, persistent TLRs activation can alter the stability of Tregs, leading to their reduced ability to suppress autoimmunity. While TLRs signaling is essential for initiating immune responses, it can suppress Treg differentiation in certain contexts. For example, in the presence of strong TLRs’ signals, such as those induced by infection or inflammation, the induction of Tregs is reduced. This phenomenon has been observed in diseases like SLE (12), where TLRs signaling not only drives the activation of autoreactive T and B cells but also diminishes Treg numbers and function. A critical aspect of immune regulation is the balance between Th17 cells and Tregs (75). TLRs signaling can shift this balance towards Th17 cell dominance by promoting the differentiation and expansion of Th17 cells at the expense of Tregs (68). This shift is particularly relevant in autoimmune diseases, where an increase in Th17 cells and a decrease in Treg numbers contribute to the loss of immune tolerance and tissue damage.

#### 3.4.3 Impact of TLRs signaling on immune tolerance and autoimmunity

The dysregulation of TLRs signaling, particularly its ability to promote Th17 differentiation and inhibit Treg function, can have profound consequences for immune tolerance. By skewing the immune response towards Th17 cells, TLRs signaling can disrupt the delicate balance that maintains tolerance to self-antigens. This disruption can lead to the development of autoimmune diseases, where the immune system attacks the body’s own tissues. TLRs activation enhances Th17 responses, leading to increased production of IL-17 and other inflammatory cytokines (69). This contributes to chronic inflammation, tissue damage, and autoimmunity. In diseases such as RA, psoriasis, and MS, Th17-driven inflammation is a key feature of disease pathology. The failure of Tregs to maintain tolerance, due to TLRs-induced cytokine imbalances, allows autoreactive T cells to escape regulation (76). This results in the loss of self-tolerance and the development of autoimmune diseases.

TLRs signaling plays a critical role in regulating T cell responses, particularly by promoting the differentiation of Th17 cells and potentially inhibiting Treg function. This dysregulation of the Th17/Treg balance can lead to the breakdown of immune tolerance, contributing to autoimmune diseases. Targeting TLRs pathways, modulating cytokine signaling, and restoring Treg function may provide new therapeutic strategies for treating autoimmune conditions driven by an imbalance in T cell responses.

### 3.5 Regulation of TLRs expression

The regulation of TLRs expression in MG involves multiple interconnected mechanisms that establish a pro-inflammatory positive feedback loop. Primarily, the predominant cytokine milieu in MG - particularly interleukin-6 (IL-6) and tumor necrosis factor-alpha (TNF-α) (77) - can directly upregulate TLRs’ expression (78), creating a self-perpetuating cycle of inflammation. Furthermore, dysregulated post-transcriptional control by microRNAs (miRNAs), notably altered miR-146a levels that target TLRs signaling pathways, contributes significantly to disease pathogenesis (41). Additionally, aberrant epigenetic modifications, including DNA methylation and histone alterations, lead to upregulated expression of specific TLRs genes, exacerbating immune dysregulation (79). These interrelated mechanisms collectively sustain the characteristic chronic inflammatory state observed in MG.

## 4 Genetic variations in TLRs pathways and MG susceptibility

TLRs pathways play a critical role in shaping individual immune responses, predisposing certain individuals to autoimmune diseases, including MG. TLRs are key players in the innate immune system, bridging the gap between innate and adaptive immunity. Any polymorphism or epigenetic alteration affecting TLRs genes or their downstream signaling molecules can disrupt immune tolerance, leading to the development of autoimmunity. This section delves into the specific genetic variations in TLRs pathways that may contribute to MG susceptibility, focusing on single nucleotide polymorphisms (SNPs), epigenetic modifications, and their functional implications.

### 4.1 SNPs in TLRs genes

SNPs in TLRs genes have been extensively studied in the context of autoimmune diseases (80). In MG, these genetic variants may influence the expression and function of TLRs, leading to aberrant immune activation and inflammation.

#### 4.1.1 TLR3 polymorphisms

TLR3 detects double-stranded RNA (dsRNA) from viral infections (21). It plays a role in regulating type IIFN responses, which are linked to autoimmunity (31). TLR3 gene missense mutation has been linked to susceptibility to autoimmune diseases, potentially by enhancing type I IFN signaling pathways (81). Given that TLR3 missense mutations have been linked to autoimmune susceptibility in conditions like MS (81), it is plausible that such polymorphisms also contribute to the pathogenesis of MG, although a direct association in MG remains to be conclusively established.

#### 4.1.2 TLR4 polymorphisms

TLR4 is critical for recognizing bacterial LPS and initiating inflammatory responses (19). Dysregulation of TLR4 signaling can result in a heightened inflammatory state, contributing to autoimmunity. SNPs such as Asp299Gly and Thr399Ile in the TLR4 gene have been associated with altered receptor function. These variants can modify the ability of TLR4 to respond to LPS and endogenous ligands, potentially influencing MG susceptibility. The Asp299Gly and Thr399Ile variants have been associated with altered LPS responsiveness and may influence MG risk (82). The heightened inflammatory response due to these variants may exacerbate the autoimmune attack on NMJ components.

#### 4.1.3 TLR9 polymorphisms

TLR9 recognizes unmethylated CpG DNA motifs (23), which are commonly found in bacterial and viral DNA. It is also implicated in recognizing endogenous DNA released during cell damage or stress (83). The TLR9 gene variant -1237T/C has been associated with altered receptor expression and function. This variant may enhance the recognition of self-DNA, leading to increased autoantibody production in MG. Altered TLR9 activity can stimulate autoreactive B cells, promoting the production of pathogenic antibodies such as anti-AChR and anti-MuSK (84), which are hallmarks of MG.

#### 4.1.4 Other TLRs gene polymorphisms

Variants in other TLRs genes, including TLR2 (27) and TLR7 (85), have been associated with autoimmune diseases and may contribute to MG susceptibility. For example, TLR7 polymorphisms, particularly in female patients (due to the X-chromosome location of TLR7), may lead to increased autoantibody production and disease severity (86).

### 4.2 Variations in downstream TLRs signaling molecules

SNPs in genes encoding adaptor proteins and signaling molecules downstream of TLRs can also impact immune responses in MG.

#### 4.2.1 MyD88 polymorphisms

MyD88 is a central adaptor protein for most TLRs, mediating pro-inflammatory signaling pathways (87). Variants in the MyD88 gene may impair the regulation of inflammatory cytokines (88), such as IL-1β and IL-6, which are elevated in MG patients. Dysregulated MyD88 signaling could lead to excessive activation of autoreactive T and B cells, contributing to autoimmunity.

#### 4.2.2 TRIF polymorphisms

TRIF mediates TLR3 and TLR4 signaling, particularly pathways leading to type I IFN production. Genetic variants in the TRIF gene may enhance type I IFN responses (89), promoting chronic inflammation in the thymus and peripheral tissues in MG.

#### 4.2.3 IRAKs and TRAF6 polymorphisms

IRAKs (IL-1 receptor-associated kinases) and TRAF6 are key molecules in TLRs signaling pathways (90). Variants in these genes may disrupt downstream signaling, leading to prolonged NF-κB activation and excessive cytokine production (91), which are characteristic features of MG-related inflammation.

### 4.3 Epigenetic regulation of TLRs genes

Epigenetic modifications, including DNA methylation and histone acetylation, play a crucial role in regulating TLRs expression and function (79). Aberrant epigenetic changes, such as altered epigenetic profiles in thymic tissues and PBMCs, may influence MG susceptibility by altering TLRs-mediated immune responses.

#### 4.3.1 DNA methylation

DNA methylation in the promoter regions of TLRs genes can suppress their expression (92), while hypomethylation can lead to overexpression (93). Studies have demonstrated hypomethylation of TLR4 and TLR9 promoters in autoimmune diseases. Similar changes in MG patients may result in heightened TLRs activity, contributing to the breakdown of immune tolerance.

#### 4.3.2 Histone modifications

Histone acetylation and deacetylation regulate chromatin accessibility and gene expression (94). Dysregulated histone acetylation in TLRs genes has been observed in thymic tissues of MG patients, potentially enhancing local inflammation and autoreactive T-cell activation.

#### 4.3.3 Non-coding RNAs

MiRNAs and long non-coding RNAs (lncRNAs) modulate TLRs signaling by targeting mRNAs of TLRs or their signaling molecules (95). miRNAs are small non-coding RNAs that post-transcriptionally regulate gene expression. In MG, dysregulated miRNAs have been implicated in modulating TLRs expression. For example, altered levels of miR-150-5p, miR-155, miR-146a-5p, miR-20b, miR-21-5p, miR-126, let-7a-5p, and let-7f-5p, which target TLRs signaling pathways, have been associated with disease pathogenesis (96).

### 4.4 Interaction between genetic and environmental factors

Genetic susceptibility to MG due to TLRs variations may be influenced by environmental factors (97), such as infections, stress, and smoking. For example: 1) viral infections: infections by viruses that activate TLR3, TLR7, or TLR9 may act as triggers in genetically predisposed individuals (42); 2) thymic microenvironment: environmental factors influencing the thymic microenvironment can exacerbate TLRs-mediated immune responses (36), promoting thymic hyperplasia and ectopic germinal center formation in MG patients.

## 5 Therapeutic implications of targeting TLRs in MG

Genetic variations in TLRs pathways play a critical role in shaping individual immune responses, predisposing certain individuals to autoimmune diseases, including MG. Genetic variations in TLRs pathways, including SNPs, epigenetic modifications, and downstream signaling disruptions, significantly contribute to the susceptibility and progression of MG. These variations not only affect innate immune responses but also shape the adaptive immune landscape, promoting the production of pathogenic autoantibodies and chronic inflammation. This section delves into the specific genetic variations in TLRs pathways that may contribute to MG susceptibility, focusing on SNPs, epigenetic modifications, and their functional implications. Elucidating the role of TLRs genetic variations in MG will enhance our understanding of disease mechanisms and pave the way for novel diagnostic and therapeutic strategies.

Understanding genetic variations in TLRs pathways provides valuable insights into MG pathogenesis and potential therapeutic approaches: 1) personalized medicine: identification of TLRs polymorphisms in MG patients may allow for risk stratification and personalized treatment strategies; 2) targeted therapies: TLRs inhibitors or modulators could be developed to target specific genetic variants associated with MG susceptibility; 3) future research: further studies are needed to explore the functional impact of TLRs variations in MG, using techniques such as genome-wide association studies (GWAS) and clustered regularly interspaced short palindromic repeats-associated protein 9 (CRISPR-Cas9)-based gene editing.

### 5.1 TLR antagonists and cytokine blockade

Given the central role of TLR-driven inflammation in MG, targeting these pathways holds therapeutic promise. Strategies include direct TLR inhibition and blockade of key downstream cytokines. Inhibiting specific TLRs, particularly those implicated in MG pathogenesis, represents a direct therapeutic approach. For instance, blocking TLR4 has been shown to mitigate aberrant immune activation in MG thymic tissues (98). Similarly, antagonists of TLR7 and TLR9, which are overexpressed in MG thymus and contribute to IFN and autoantibody production, have demonstrated efficacy in ameliorating disease in experimental autoimmune models (99).

Since cytokines like IL-6 and TNF-α are critical effectors downstream of TLR signaling for the survival and differentiation of autoreactive B and Th17 cells, therapies targeting these cytokines are promising. The IL-6 receptor antagonist tocilizumab has shown beneficial effects in case reports and small series of refractory MG patients (100). Similarly, the TNF-α inhibitor infliximab has been explored in MG, although its use requires careful consideration of potential exacerbations (101).

In addition to the strategies targeting specific TLRs or downstream cytokines, numerous TLRs pathway-targeting agents are under investigation in other autoimmune diseases, offering potential future directions for MG therapy. **Table 3** summarizes some TLR-targeting agents currently in the research and development stage.

**Table 3:** TLRs-targeting agents under investigation for autoimmune diseases with potential relevance to MG.

TLRs target	Agent name/type	Mechanism of action	Development stage (primary disease context)	Potential implication for MG	Reference(s)
TLR4	TAK-242	Selective small-molecule inhibitor of TLR4 signaling	Preclinical/Clinical (chronic rhinosinusitis)	May suppress TLR4-driven thymic inflammation and autoreactive lymphocyte activation.	(102, 103)
TLR4	Eritoran	TLR4 antagonist	Clinical (insulin resistance, hepatic inflammation)	May mitigate excessive TLR4 activation triggered by endogenous DAMPs or LPS analogues.	(104, 105)
TLR7/TLR8/9	IMO-8400	Antagonist of TLR7, TLR8, and TLR9	Preclinical/Clinical (dermatomyositis)	May inhibit TLR7/8/9 pathways activated by viral/self nucleic acids, reducing type I IFN and pro-inflammatory cytokine production.	(106)
TLR7	Anti-TLR7 monoclonal antibodies	Blocks TLR7 activation	Preclinical (SLE)	Particularly relevant for MG patients with high TLR7 expression or gain-of-function variants.	(107)
TLR9	CpG-ODN	TLR9 antagonist	Preclinical (IgA nephropathy)	May inhibit the production of aberrantly glycosylated inclusion.	(108)
TLR9	E6446	TLR9 antagonist	Preclinical (right ventricular dysfunction)	May improve macrophage infiltration and fibrosis.	(109)
MyD88	TJ-M2010-2	MyD88 dimerization inhibitor	Preclinical (breast cancer)	Broadly inhibits MyD88-dependent TLR signaling (TLR2,4,5,7,8,9), potentially controlling aberrant activation of multiple TLRs more effectively.	(110)
TRIF	No highly specific potent inhibitor available	Interferes with TRIF-dependent pathway	Early research	Theoretically could specifically modulate TLR3/4-driven type I IFN responses, but requires further development.	/

TLRs, Toll-like receptors; MG, myasthenia gravis; SLE, systemic lupus erythematosus; DAMPs, damage-associated molecular patterns; LPS, lipopolysaccharide; IFN, interferon; ODN, oligodeoxynucleotide; MyD88, myeloid differentiation primary response 88; TRIF, TIR-domain-containing adapter-inducing interferon-β.

### 5.2 LR4 recognizes LPS modulation of antigen-specific responses

Emerging strategies aim to modulate the immune system to induce tolerance specifically to autoantigens like AChR. Preclinical studies suggest that TLR9-stimulated pDCs can produce both antiviral (IFN-β) and pro-inflammatory (IL-6) cytokines, highlighting their complex role in shaping immune responses in experimental autoimmune settings (111). Leveraging this knowledge, the potential of incorporating TLR ligands into antigen-specific tolerance protocols represents a novel, though still experimental, avenue for restoring immune homeostasis in MG.

## 6 Biomarker potential and personalized medicine

The deepened understanding of the role of TLRs in MG not only reveals new therapeutic targets but also advances their potential application in biomarker development and personalized medicine. TLR expression levels, signaling pathway activity, and genetic variations hold promise as clinically useful biomarkers.

Studies have shown that the expression levels of specific TLRs (e.g., TLR9) positively correlate with QMG scores (49). Therefore, monitoring the TLR expression profile in PBMCs or specific immune cell subsets could aid in the objective assessment of disease activity. Furthermore, monitoring levels of key downstream cytokines driven by TLRs (e.g., IL-6, TNF-α) might provide information on the inflammatory state and disease severity. Genetic polymorphisms in the TLR pathway (e.g., SNPs in TLR7, TLR9) may partially explain the clinical heterogeneity and varying treatment responses among MG patients (86). Genotyping patients for their TLR-related genetic background could potentially guide future treatment choices. For instance, patients carrying variants associated with TLR7 overactivity might respond better to TLR7 antagonist therapy. Similarly, refractory MG patients with significantly elevated serum IL-6 levels might be more specifically targeted with IL-6 receptor antagonists like tocilizumab (100). Drugs directly targeting TLRs or their downstream signaling molecules (see **Table 3**) are under development. Identifying which patient subgroups are most likely to benefit from these specific interventions is crucial. Pre-treatment analysis of TLR expression profiles in patient thymic tissue or peripheral immune cells could help stratify patients most suitable for corresponding TLR-targeted therapies. Concurrently, dynamic changes in TLR pathway activity during treatment could serve as pharmacodynamic biomarkers for assessing drug efficacy.

In conclusion, integrating TLR biology with clinical characteristics holds the potential for precise stratification of MG in the future, enabling the selection of the most effective treatment strategies based on the specific immunopathological mechanisms of individual patients, thereby moving towards a new era of personalized medicine.

## 7 Future directions

Epigenetic therapies, while currently lacking direct evidence in MG, represent a promising future direction based on their mechanism of action. Aberrant DNA methylation and histone modifications have been implicated in the dysregulation of TLR genes in other autoimmune diseases. Building on findings from other autoimmune diseases where aberrant DNA methylation and histone modifications dysregulate TLR genes, agents such as DNA methyltransferase inhibitors (e.g., 5-azacytidine) and histone deacetylase (HDAC) inhibitors (e.g., entinostat) could potentially restore balanced TLR expression (112). Combination strategies targeting both epigenetic layers may synergistically reset pathogenic TLR networks. The development of cell-specific delivery systems (e.g., nanoparticle-based targeting of B cells or thymic epithelial cells) will be crucial to maximize efficacy and minimize off-target effects (113).

Furthermore, the role of TLR polymorphisms in MG requires systematic investigation through large-scale genetic association studies to identify MG-specific SNPs, followed by functional analyses to determine their impact on TLR signaling and contribution to disease pathogenesis.

## 8 Conclusion

TLRs play a multifaceted and central role in the pathogenesis of myasthenia gravis, driving the innate and adaptive immune responses that lead to autoantibody production, chronic inflammation, and subsequent tissue damage. Our understanding of TLRs dysregulation provides profound insights into MG pathogenesis and unveils novel potential targets for therapeutic intervention. Future research must prioritize elucidating the precise contributions of individual TLRs and their signaling pathways in MG, and rigorously evaluate the efficacy of TLRs-targeted therapies in both preclinical and clinical settings. Although direct evidence linking TLRs polymorphisms to MG remains limited, their established role in immune regulation and autoimmunity strongly suggests that genetic variations in these receptors influence disease susceptibility, meriting further exploration.

## Glossary

MG: myasthenia gravis
NMJ: neuromuscular junction
AChR: acetylcholine receptor
MuSK: muscle-specific kinase
LRP4: low-density lipoprotein receptor-related protein 4
TLRs: toll-like receptors
PRRs: pattern recognition receptors
PAMPs: pathogen-associated molecular patterns
DAMPs: damage-associated molecular patterns
DCs: dendritic cells
IFN: interferon
SLE: systemic lupus erythematosus
RA: rheumatoid arthritis
T1D: type 1 diabetes
MyD88: myeloid differentiation primary response 8
TIR: toll/interleukin-1 receptor
TRIF: TIR-domain-containing adapter-inducing interferon-β
IRAKs: interleukin-1 receptor-associated kinases
TRAF6: TNF receptor-associated factor 6
NF-κB: nuclear factor kappa-light-chain-enhancer of activated B cells
AP-1: activator protein 1
IRFs: interferon regulatory factors
LPS: lipopolysaccharides
LRR: leucine-rich repeat
TIRAP/Mal: TIR domain-containing adaptor protein
TAB1: TGF-β
TAB2: TGF-β
TAK1: TGF-β
IKK: inhibitor of nuclear factor kappa-B kinase
JNK: c-Jun N-terminal kinase
MAPK: mitogen-activated protein kinase
TBK1: TANK-binding kinase 1
APCs: antigen-presenting cells
NK: natural killer
TECs: thymic epithelial cells
SOCS: suppressors of cytokine signaling
JAK-STAT: janus kinase-signal transducer and activator of transcription
HMGB1: high mobility group box1
miR-146a: microRNA-146a
PBMCs: peripheral blood mononuclear cells
TECs: thymic epithelial cells
cTECs: cortical TECs
mTECs: medullary TECs
AIRE: Autoimmune Regulator
pDCs: plasmacytoid dendritic cells
QMG: Quantitative MG
EAMG: experimental autoimmune myasthenia gravis
ssRNA: single-stranded RNA
EBV: Epstein-Barr virus
dsRNA: double-stranded RNA
WNV: West Nile virus
COVID-19: coronavirus disease 2019
CMV: cytomegalovirus
HCV: hepatitis C virus
HIV: human immunodeficiency virus
VZV: varicella-zoster virus
HSV-1: herpes simplex virus type 1
HSV-2: herpes simplex virus type 2
PV: poliovirus
ZV: Zika virus
DENV: dengue virus
HTLV-1: human T-lymphotropic virus type 1
TGF-β: transforming growth factor beta
Th1: T helper 1
Th17: T helper 17
RORγt: retinoic acid receptor-related orphan receptor gamma t
MS: multiple sclerosis
Tregs: regulatory T cells
Foxp3: forkhead box P3
SNPs: single nucleotide polymorphisms
GWAS: genome-wide association studies
CRISPR-Cas9: clustered regularly interspaced short palindromic repeats-associated protein 9
IL: interleukin
TNF-α: tumor necrosis factor alpha
IFN-β: interferon beta
IFN-γ: interferon gamma
STAT3: signal transducer and activator of transcription 3
miRNA: microRNA
lncRNA: long non-coding RNA
HDAC: histone deacetylase
TCR: T cell receptor
pDCs: plasmacytoid DCs
IRAK1: interleukin-1 receptor-associated kinase 1
